# Risk Factors for Persistent Shoulder Pain After Cervical Spine Surgery

**DOI:** 10.1111/os.12531

**Published:** 2019-10-29

**Authors:** Erica Kholinne, Jae‐Man Kwak, Yucheng Sun, Hyun‐Joo Lee, Kyoung Hwan Koh, In‐Ho Jeon

**Affiliations:** ^1^ Department of Orthopedic Surgery St. Carolus Hospital Jakarta Indonesia; ^2^ Department of Orthopaedic Surgery, Asan Medical Center, School of Medicine University of Ulsan Ulsan South Korea; ^3^ Department of Hand Surgery, Affiliated Hospital of Nantong University Nantong, Nantong University Jiangsu China; ^4^ Department of Orthopaedic Surgery Kyungpook National University Hospital Daegu South Korea

**Keywords:** Cervical vertebrae, Risk factors, Shoulder pain, Spondylosis

## Abstract

**Objective:**

To define risk factors of having persistent shoulder pain following cervical spine surgery.

**Methods:**

From April 1995 to May 2012, 862 patients underwent cervical spine surgery in a tertiary referral university hospital. Ninety‐six patients were referred to a shoulder surgeon for persistent shoulder pain over 3 months after cervical spine surgery. Thirty‐five were excluded from the study due to failure to follow‐up or lack of radiographic data. We analyzed a total of 61 patients as patient group (PG) compared to age, sex, and surgeon matched control group (CG) with no shoulder pain after surgery. Medical records were reviewed for age, sex, level of cervical surgery, approach of cervical surgery, underlying medical problems, final diagnosis of the shoulder pathology, and shoulder visual analog scale (VAS) score. The pre‐ and post‐operative variables for level of cervical surgery, approach of cervical surgery, underlying medical history, and shoulder VAS were compared and analyzed in this study.

**Results:**

The number of patients with lower cervical level surgery was significantly higher (91 patients, 74.6%) compared to upper cervical level surgery (31 patients, 25.4%) (*P* = 0.005). Anterior approach was more frequently used (70 patients, 57.3%) compared to posterior approach (52 patients, 42.7%) (*P* < 0.001). The final diagnosis of shoulder pathology in PG were rotator cuff disease in 31 patients, adhesive capsulitis in 18 patients, and calcific tendinitis in 10 patients respectively. No significant difference for preoperative shoulder VAS score was found in both groups. A lower level of cervical spine pathology in patients (C_4_‐T_1_ level) and posterior approach surgery were factors that significantly presented with more shoulder pain. Subgroup analysis revealed no statistical difference for level of cervical surgery and approach of cervical surgery among groups with different shoulder pathology.

**Conclusions:**

The current study includes patients with persistent shoulder pain following cervical surgery without having evidence of concurrent preexisting shoulder pathology documented previously. We suggest that posterior approach and lower level of cervical surgery patients could be clinically relevant risk factors for persistent shoulder pain following cervical spine surgery.

## Introduction

Cervical spondylosis is one of the most common degenerative spine disorders which typically manifests as posterior neck and shoulder pain.^1^, ^2^, ^3^, ^4^, ^5^Cervical spine and shoulder disorders frequently have very similar presentations and can be difficult to differentiate.^3^, ^6^, ^7^ Cervical spondylosis not only results in posterior neck pain but can also cause shoulder pain that can be confused with shoulder disorder with rotator cuff pathology as the most common pathology.^6^ However, with a careful history, physical exam, and imaging studies, the true source of a patient's symptoms can be defined and treated.^3^, ^6^

Cervical spine decompression surgery has been frequently performed to gain neurologic recovery and pain reduction to improve functional outcome.^4^, ^5^, ^8^ However, persistent pain following cervical spine surgery is not uncommon.^3^, ^4^, ^5^, ^8^ Studies have reported that one‐forth of patients still have persistent posterior neck and shoulder pain following laminoplasty decompression surgery.^4^, ^5^ A systematic review for axial neck pain after posterior cervical spine surgery described that laminoplasty can aggravate axial neck pain and suggests discs, muscles, facet joints, and nerves as the source of the pain; however, there was no data on its relationship with specific shoulder disorder confirmed.^8^ Another study showed that 38.4% of the included 13 patients who underwent concurrent anterior cervical spine fusion and shoulder procedure (subacromial decompressions or rotator cuff repairs) had more significant shoulder pain compare to preoperative condition following surgeries^3^. Regardless, there is a scarcity of information on this topic, and the present literature only provides general discussion of having persistent pain following a specific type of cervical spine surgery without specific analysis of the cervical spine surgical level and surgical approach.

The purposes of the current study were: (i) to review and analyze the risk factors of having persistent shoulder pain after cervical spine surgery; (ii) to analyze the relationship between the level of cervical spine to persistent shoulder pain; and (iii) to analyze the relationship between the surgical approach and persistent shoulder pain. We hypothesize that lower levels of cervical spine pathology and posterior surgical approach could contribute as risk factors toward persistent shoulder pain.

## Materials and Methods

### *Inclusion and Exclusion Criteria*

The study reviewed patients who underwent cervical spine surgery in a tertiary referral university hospital from April 1995 to May 2012. A total of 862 patients were collected from a medical data bank. Ninety‐six patients were referred to a shoulder surgeon for persistent shoulder pain over 3 months after cervical spine surgery. Inclusion criteria was made according to the PICO (Patient, Intervention, Comparison, and Outcome) principle as follows: (i) Patients: skeletally mature (over 18 years of age) with documented neurophysiological study prior to cervical spine surgery; (ii) Intervention: persistent shoulder pain despite conservative measures following cervical spine surgery; (iii) Comparison: patient with matching age, sex, and operating spine surgeon without persistent shoulder pain following cervical spine surgery; and (iv) Outcome: level of cervical surgery and approach of cervical surgery. Exclusion criteria were as follows: (i) skeletally immature; (ii) history of trauma and surgery to the affected shoulder; and (iii) lack of radiographic data.

### *Study Design*

Retrospective review was done for medical records. Medical data were reviewed for age, sex, level of cervical surgery, approach of cervical surgery, underlying medical history, shoulder visual analogue scale (VAS) before and after cervical spine surgery, and the final diagnosis of the shoulder pathology made by the shoulder surgeon. Sixty‐one patients (30 male, 31 female) were finally included in the study defined as patient group (PG). The preoperative information regarding concurrent preexisting shoulder pathology was not described in the medical record. All patients finished standard shoulder examination by a single, senior shoulder surgeon. Plain radiograph of shoulder (anteroposterior, axillary lateral, and outlet views) and ultrasonography were used for additional diagnostic tool. Another 61 patients with matching age, sex, and operating spine surgeon with no shoulder pain after surgery were selected from the data bank as control group (CG).

### *Outcome Measures*

#### *Level of Cervical Surgery*

The level of cervical surgery was categorized as upper and lower cervical. Upper cervical level includes C_1‐3_, and lower cervical level includes C_4_‐T_1_. The level of cervical surgery was recorded as per described in the operation record.

#### *Approach of Cervical Surgery*

The approach of cervical surgery was defined as anterior, posterior, and combined anterior–posterior approach. The approach of cervical surgery was recorded as per described in the operation record.

#### *Underlying Medical History*

Underlying medical history was categorized as diabetes, other peripheral nerve disease, peripheral vascular disease, and brain lesion. Underlying medical history was recorded as per described in the medical record since the first outpatient visitation related to the cervical spine surgery.

#### *Shoulder Visual Analogue Scale*

Shoulder VAS included both before and after cervical spine surgery. The VAS scoring system was a continuous single scale item which was anchored by two verbal descriptors, one for each symptom extreme. Response for pain intensity will be anchored by “no pain” (score of 0) and “worst imaginable pain” (score of 10). The minimum clinical importance difference of VAS was set at 2.5 based on previous study.^9^

### *Statistical Analysis*

The Kolmogorov–Smirnov test was used for normality distribution. All data showed skewed distribution, therefore non‐parametric statistic tests were used, and data was expressed with median and interquartile range (IQR). The pre‐ and post‐operative variables for level of cervical surgery, approach of cervical surgery, and underlying medical history, and shoulder VAS were compared and analyzed with Wilcoxon signed‐rank test. The differences regarding level of cervical surgery and approach of cervical surgery among each shoulder pathology group were compared with Kruskal Wallis test. *P*‐value was set at <0.05 to be significant. All statistical testing was performed using SPSS version 17 for Windows (SPSS, Inc., Chicago, IL, USA).

## Results

### *Demographic Data*

Baseline characteristic data was shown in Table 1. The number of patients with lower cervical level surgery was significantly higher (91 patients, 74.6%) compared to upper cervical level surgery (31 patients, 25.4%) (*P* = 0.005). Anterior approach was more frequently used (70 patients, 57.3%) compared to posterior approach (52 patients, 42.7%) (*P* < 0.001).

**Table 1:** Baseline characteristics of study population

Variables		n	Percent (%)	Mean	SD
Group	Control group	61	50	—	—
	Patient group	61	50	—	—
Sex	M	60	49.2	—	—
	F	62	50.8	—	—
Age		122	—	52.60	12.14
Location of surgery	Lower cervical	91	74.6	—	—
	Upper cervical	31	25.4	—	—
Approach of cervical spine operation	Anterior	70	57.4	—	—
	Posterior	50	41.0	—	—
	Combined	2	1.6	—	—
preoperative VAS score		122	—	4.73	2.29
postoperative VAS score		122	—	2.63	2.10
VAS difference		122	—	2.10	2.13

F, female; M, male; SD, Standard Deviation; VAS, Visual Analog Scale.

### *Cervical Surgery Characteristics*

Table 2 demonstrated the comparison of level of cervical surgery, approach of cervical surgery, and underlying medical history and shoulder VAS between CG and PG. The number of the patients with lower cervical spine level surgery was found significantly higher in PG (*P* = 0.005) compared to CG. The number of the patients with posterior approach for cervical surgery was found significantly higher in PG (*P* < 0.001). Thirty‐five patients (58.4%) from PG had posterior surgical approach: 12 had laminoplasty and 23 had laminectomy. Twenty‐six patients (42.6%) had anterior surgical approach: Anterior Cervical Corpectomy and Fusion (ACCF) in six patients, Anterior Cervical Discectomy and Fusion (ACDF) in six patients, and Artificial Disc Replacement (ADR) in 12 patients. Eighty‐six percent of all patients had no underlying medical history as described.

**Table 2:** Comparison of cervical surgery characteristic and shoulder VAS score between control and patient group

Variables	Sub variables	Control Group (n = 61)			Patient Group (n = 61)			*P*‐value
		n	Percent (%)	Median, IQR	n	Percent (%)	Median, IQR	
Level of cervical surgery	Lower	34	55.7	—	57	93.4	—	0.005*
	Upper	27	44.3	—	4	6.6	—	
Approach of cervical surgery	Anterior	44	72.1	—	26	42.6	—	<0.001*
	Posterior	17	27.9	—	35	58.4	—	
Underlying medical history	None	54	88.5	—	51	83.6	—	0.581
	Positive	7	11.5	—	10	16.4	—	
Pre‐operation VAS		4	—	(3, 5.5)	6	—	(3, 6)	0.496
Post‐operation VAS		2	—	(0, 3)	3	—	(2, 5)	<0.001*
VAS difference		3	—	(1, 4)	1	—	(0, 2)	0.002*

* Significant *P*‐value (*P* < 0.05).

IQR, Interquartile Range; VAS, Visual Analog Scale.

### *Shoulder Visual Analogue Scale (VAS) Characteristics*

There was no significant difference regarding underlying medical history between CG and PG. No significant difference of shoulder pain before cervical spine surgery was found between two groups (*P* = 0.496). Shoulder VAS improved from an average of 6 (range 4–7) to 3 (range 2–6) postoperatively in PG. However, severity of postoperative shoulder pain was more profound with less improvement of pain after surgery in PG (*P* < 0.001, *P* = 0.002).

### *Subgroup Analysis for Shoulder Pathology*

Table 3 described the subgroup analysis for each final diagnosis of shoulder pathology. Final diagnosis of shoulder pathology was rotator cuff disease in 31 patients (50.8%), adhesive capsulitis in 18 patients (29.5%), and calcific tendinitis in 10 patients (16.3%). Other pathology included peripheral neuropathy and incomplete fracture in two patients (1.6%). There was no significant association between the shoulder pathology with the level of cervical spine surgery (*P* = 0.465) and with the type of cervical spine approach (*P* = 0.226).

**Table 3:** Subgroup analysis for each final diagnosis of shoulder pathology in patient group

Variables	Diagnosis	Rotator cuff disease (n = 31)		Calcific tendinitis (n = 10)		Adhesive capsulitis (n = 18)		Others (n = 2)		*P*‐value
		n	Percent (%)	n	Percent (%)	n	Percent (%)	n	Percent (%)	
Cervical Level of surgery	Lower	30	96.7	8	80.0	17	94.5	2	100.0	0.465
	Upper	1	3.2	2	20.0	1	5.5	0	0.0	
Approach	Anterior	14	45.2	3	30.0	9	50.0	0	0.0	0.226
	Posterior	17	54.8	7	70.0	9	50.0	2	100.0	
	Combined	4	12.9	2	20.0	4	77.8	0	0.0	

## Discussion

Persistent shoulder pain after cervical surgery remains devastating for most of our patients. Our study shows that persistent pain after cervical spine surgery is not uncommon. Various shoulder joint pathologies have been identified when examined by a shoulder surgeon. Radicular pain, including axial neck pain from cervical spine disorders, may mask the diagnosis of concomitant shoulder disorder. From the spine surgeon's point of view, better understanding of this condition is important because the persistent shoulder pain related to shoulder joint pathology is complicated to explore before the surgery. In this study, patients who had persistent shoulder pain after cervical spine operation revealed various shoulder joint disorders. There have not been many studies on patients with persistent shoulder pain after cervical spine surgery.

Studies have reported that neck and shoulder pain, termed as axial pain, is presented after cervical laminoplasty.^4^ For about 25% of post‐laminoplasty patients, axial pain remains a continuous chief complaint after surgery.^5^ Nevertheless, cases were only limited to cervical laminoplasty. Persistent axial pain was postulated to arise from the detachment of muscle insertion at C_7_ spinous process. Hence, the axial pain merely increased in sitting position rather than in supine position. Furthermore, there was no investigation to rule out concurrent shoulder maladies.

In our study, the persistent pain that developed post cervical spine surgery was not affected by position. Patients who complained of persistent shoulder pain were more profound in lower cervical surgery group compared to the control group. Nevertheless, we are unable to draw a cogent conclusion as lower cervical surgery will be a relevant risk factor to persistent shoulder pain. This is because most common cervical disc pathologies and spondylosis were located at the level of C_5‐7._ 1, 10 Surgical approach to the posterior cervical spine will involve detachment of muscles insertion to its spinous process. Spinous process of the cervicothoracic junction contributes as fulcrums for shoulder suspensory muscle. To disturb such biomechanics will tenaciously increase the load and strain to each muscle belly of the shoulder suspensory complex. Therefore, muscle complex becomes susceptible to fatigue resulted in lower pain threshold. Studies also reported the relationship between surgical approach in the cervical spine and its risk factors for axial neck pain.^8^ They concluded that posterior surgical approach is a risk factor for aggravating axial neck pain. Our study also supports the conclusion in the patients with persistent shoulder pain, as we found more patients underwent surgery through posterior approach compared to the control group.

The most common diagnosis of the shoulder joint pathology was rotator cuff disease, followed by adhesive capsulitis and calcific tendinitis. We found no statistical difference in each group in terms of clinical variables. Thus, when patients complain of persistent shoulder pain after cervical spine surgery the existence of these shoulder pathologies should be kept in mind as a potential cause.

The current study includes patients who underwent cervical surgery without having evidence of concurrent preexisting shoulder pathology documented previously. To date, there is no consensus to treat patients with both cervical spine and shoulder pathology. We postulate that the shoulder pathology was an oversight due to the agonizing and dominant symptoms of the cervical pathology, hence, the patient may have neglected any minor symptoms arising from the shoulder. A thorough investigation must be initiated to determine whether the pain comes primarily from the cervical spine, shoulder, or both for surgical strategy. If pain is predominantly originating from the shoulder, we suggest approaching the shoulder first and giving careful observation to the cervical pathology and *vice versa*. For complex patients with equal symptoms, we suggest initiating surgery for both aspects. One study did describe that perhaps two procedures are not necessary.^3^ One consideration is that shoulder procedure is usually a lesser procedure than the other.

### *Limitations*

Our study had several limitations. Firstly, the number of subjects is relatively small, and the study was retrospectively designed. Secondly, we did not include cervical symptoms (radiculopathy or myelopathy) and indication for cervical surgery approach as study variables. We specifically retrieved data from the referred patients to the shoulder surgeon. We recognized that this could be a potential bias. Nonetheless, this study analyzed the biggest number of patients with control groups compared to the literature reported, and a single shoulder surgeon conducted all examinations, thus the diagnosis protocol was consistent.

As there have only been a few studies reporting shoulder pain after cervical spine surgery, this study provides valuable clinical implications for treating patients with persistent shoulder pain after cervical spine surgery.

Our study concluded that when spine surgeons encounter lower cervical spine disorder with shoulder pain, concomitant shoulder disorder should be carefully assessed by history and physical examination of shoulder joint. Patient who present with persistent shoulder pain after cervical spine surgery had higher chance of having concurrent shoulder pathology. We suggest that posterior surgical approach may be a relevant risk factor for persistent shoulder pain following cervical spine surgery.
